# MTA Matrix Technique: Restoration of Teeth with Deep Subgingival Defects Extending Down to the Osseous Crest

**DOI:** 10.3290/j.jad.b3146843

**Published:** 2022-06-20

**Authors:** Johannes Mente, Fabian Hieber, Caroline Sekundo, Dorothee Laura Schuessler, Holger Gehrig

**Affiliations:** a Professor and Head, Division of Endodontics and Dental Traumatology, Department of Conservative Dentistry, University Hospital Heidelberg, Germany. Conceptualization, methodology, investigation, supervision, resources, wrote first draft.; b Dentist, Specialized Endodontic Practice “Dres. Hartmann, Zirkel & Kollegen”, Cologne, Germany. Investigation; wrote, reviewed, and edited the manuscript.; c Dentist, Department of Conservative Dentistry, Division of Preventive and Restorative Dentistry, University Hospital Heidelberg, Germany. Investigation; wrote, reviewed, and edited the manuscript.; d Dentist, Department of Orthodontics and Dentofacial Orthopaedics, University Hospital Heidelberg, Germany. Investigation; wrote, reviewed, and edited the manuscript.; e Dentist, Division of Endodontics and Dental Traumatology, Department of Conservative Dentistry, University Hospital Heidelberg, Germany; Specialized Endodontics and Peridontics Practice, Kandel, Germany. Conceptualization, methodology, investigation, supervision, resources, wrote first draft.

**Keywords:** MTA matrix technique, restoration of deep cavities, treatment technique, supracrestal tissue attachment, biological width, deep subgingival hard tissue defects, mineral trioxide aggregate.

## Abstract

**Purpose:**

To present a new restorative technique for the restoration of teeth with deep subgingival hard tissue defects extending down to the osseous crest without additional surgical or orthodontic interventions by combining mineral trioxide aggregate (MTA) and composite material.

**Materials and Methods:**

The MTA matrix technique starts by deeply inserting a metal matrix as far down to the bone level as possible. The matrix should then be fixated with a matrix holder in its end position. If the matrix band does not seal tightly in the deepest area of the cavity, small portions of MTA are carefully applied to the lower end of the inner side of the matrix band. The MTA acts as a barrier for fluid control. Additional haemostasis is not necessary. Subsequently, the tooth is restored with an etch-and-rinse adhesive and composite resin. The clinical effects were observed in a case series of three patients over a period of 3 to 4.5 years.

**Results:**

Excellent outcomes were observed clinically and radiologically. Teeth restored with the MTA matrix technique showed no failures due to the materials used or due to secondary caries or periodontal inflammation after an observation period of 3 to 4.5 years. Probing depths ranged from 2 to 4 mm without bleeding on probing, including the subgingivally restored areas.

**Conclusion:**

Although only a few casuistic observations are available to date, by using the MTA matrix technique, successful restoration of teeth with subgingival defects down to the alveolar bone crest seems possible without the need of additional surgical or orthodontic measures. Further clinical studies are necessary to confirm the feasibility of this technique.

A tooth with extensive crown-root destruction reaching into the epicrestal area, as depicted in Fig 1a, can present with a variety of signs and symptoms, depending on the pulpal or periodontal involvement. Microorganisms can infiltrate this area from the oral cavity, resulting in localized periodontal inflammation. This can manifest as localized attachment loss and bleeding on probing (BOP) (Fig 1a).

**Fig 1a:**
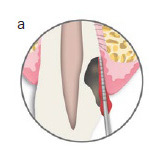
Schematic illustrating a tooth with a resorptive or carious lesion reaching down to the bone level. Typically, periodontal probing into this area results in bleeding on probing (BOP).

The restoration of such defects requires dry conditions, particularly when using adhesive materials such as composite resin. Blood or sulcular fluid infiltration must thus be avoided. As a result, deep subgingival hard tissue defects caused by non-restorable caries, external cervical root resorption, or deep cusp fractures are important reasons for the failure of vital and endodontically treated teeth.^38,40,42^

Even if restoration placement succeeds in such an extensively damaged tooth, the lower end of the restoration margin is often located very deeply within the soft tissue of the dentogingival junction close to the alveolar bone crest. The supracrestal tissue attachment (STA), formerly termed biological width, is composed of the junctional epithelium and supracrestal connective tissue attachment.^17^ Violation of STA is reported to lead to gingival inflammation, loss of periodontal attachment, and localized bone loss.^13,29,30^

Three treatment options are commonly used to avoid violation of STA prior to restoration: surgical crown lengthening, and orthodontic or surgical tooth extrusion. Surgical crown lengthening is often performed in cases of deep subgigival caries, considerable cervical root resorption, or after crown-root fracture.^4,15^ However, this may lead to attachment loss with undesirable esthetic results, compromise the crown-to-root ratio, or result in furcation involvement.^4,15^ An alternative approach is orthodontic tooth extrusion (forced eruption) combined with fibrotomy, ie, resection of the supracrestal attachment fibers.^16,33^ While this yields more esthetically pleasing results, it requires more effort and is more expensive than surgical crown lengthening. Surgical extrusion is a third treatment option to avoid violation of STA in the long term. Surgical extrusion is simpler and less time consuming compared to orthodontic tooth extrusion.^6^ The prognosis after surgical extrusion seems to be favorable with a low incidence of failure.^6,9,24^ A drawback of this treatment option is a slight risk of increased tooth mobility, marginal bone loss and root resorptions (predominantly nonprogressive), although the evidence from existing studies and case series is limited.^6,9^ This treatment option is predominantly practiced on single-rooted teeth after crown-root fracture.^6,9,18^

With the MTA matrix technique, the authors present a method that allows placement of a sufficient composite resin build-up in teeth with hard tissue defects extending to the osseous crest under dry conditions, with no adverse reaction due to STA violation, as an alternative to surgical crown lengthening, and orthodontic or surgical tooth extrusion.

## MATERIALS AND METHODS

### The MTA Matrix Technique Step-by-Step

After local anesthesia, any caries or resorptive tissue should be removed. Subsequently, a metallic matrix band (eg, Tofflemire, 1102/30, KerrHawe; Bioggio, Switzerland) is pressed very deeply into the sulcus of the affected tooth (Fig 1b) and immediately fixated with a corresponding matrix holder (eg, Tofflemire Matrix Band Holder, KerrHawe) to prevent coronal movement of the band. The authors prefer an M.O.D. Tofflemire matrix band, which can be customized with the help of sharp curved scissors to facilitate deep insertion into the cavity.

**Fig 1b:**
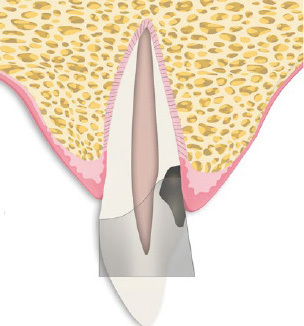
Schematic of the same tooth after removal of caries or resorptive soft tissue and insertion of a metallic matrix band.

The lower edge of the matrix band often cannot be completely adapted to the tooth (Figs 1b and 1c), leaving a small gap between it and the surrounding tissue, through which fluid or blood can seep into the restoration area. The gap is then sealed off by application of mineral trioxide aggregate (MTA) at the lower end of the matrix band to create dry conditions for cavity restoration with a light-curing filling material (Fig 1c). This method can also compensate a matrix margin that is too short in subcrestal cavities.

**Fig 1c:**
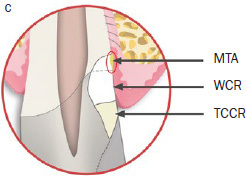
The gap between the lower margin of the matrix band and the crestal bone is sealed using a thin layer of MTA (area circled in red). Coronally and behind the MTA cement, the remaining cavity consists of white composite resin (WCR) and tooth-colored composite resin (TCCR) in the visible area of the tooth.

The MTA should preferably be applied in very small portions with an MTA gun (MAP system, yellow cannula Ø 0.90 mm, Produits Dentaires; Vevey, Switzerland). It can be compacted into the gap with a small, rounded plugger (eg, HWH 155-00, Hammacher; Solingen, Germany) or with a periodontal probe (PCPUNC 15; Hu-Friedy; Chicago, IL, USA) if the gap is very narrow. Slightly surplus MTA cement can be carefully removed with the aid of a small, sterile, moist cotton pellet. Finally, the thin MTA layer should be compressed again with the aid of this moist cotton pellet. Thus, the compressed MTA will not be washed out by the subsequent procedure of etching, rinsing, priming, and bonding, even if the MTA is layered very thinly. This allows immediate restoration of the tooth. Immediately afterwards, the dentin is etched with 37% phosphoric acid for 20 s, rinsed for at least 30 s, and then primed with a light-curing adhesive (eg, Optibond FL, Kerr; Orange, CA, USA). After this step, some layers of flowable white composite resin (WCR, eg,Tetric EvoCeram Bleach XL flow, Ivoclar Vivadent; Schaan, Liechtenstein) are applied in small portions, to make this deeply applied composite resin clearly identifiable. To this end, a flexible plastic cannula (Capillary Tip Ø 0.35 mm, Ultradent; South Jordan, Utah, USA), for instance, can be used, which can be adapted to fit on small unidose flow-composite tips. If flowable composite from larger syringes is used, metal cannulas of different diameters can also be attached to the provided luer-lock connector. A special polymerization lamp (VALO-LED curing light, Ultradent) with an adaptable fiber-optics attachment (EndoGuide lens for VALO-LED curing light) is advisable to ensure optimal light polymerization (Case 1, Fig 2e). For the visible supragingival level, a tooth-colored composite (TCCR) can be used (Fig 1c). If the applied MTA remains in place after removal of the matrix, smoothening of MTA surplus is not necessary. The authors recommend using a dental operating microscope for the whole procedure.

### Case Reports

#### Case 1

A 45-year-old male patient was referred with an irregular, asymmetric radiolucency in his first right maxillary incisor (Fig 2a). The patient reported no symptoms and all maxillary anterior teeth responded normally to cold (CO_2_) and electric pulp testing. Probing depths (PD) were <3 mm. The clinical examination showed a reddish-translucent discoloration with a small localized perforation on the palatal cervical aspect of the maxillary right central incisor (Fig 2b). Because an external cervical resorption (ECR) was suspected based on the pre-operative radiograph (Fig 2a) and the clinical signs described above, it was decided to perform a small-volume cone-beam computed tomographic (CBCT) scan (Fig 2c). On the basis of all clinical and radiographic findings, the tooth was diagnosed with external cervical resorption (class 4 according to Heithersay’s classification).^14^

**Fig 2a:**
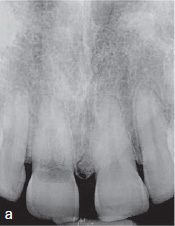
Preoperative radiograph of tooth 11.

**Fig 2b:**
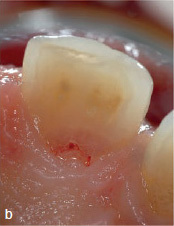
Reddish-translucent discoloration with a small localized perforation on the palatal cervical aspect of tooth 11.

**Fig 2c:**
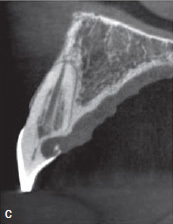
CBCT image of tooth 11 (sagittal slice) revealed a class-4 Heithersay ECR.

The patient wished to preserve the tooth. He agreed to the proposed therapy plan of removal of the resorptive tissue, endodontic treatment (ET), and restoration, as well as internal stabilization of the tooth using light-curing composite resin. The patient was informed of alternative treatments options (extraction and subsequent placement of an adhesive bridge or a dental implant).

After accessing the pulp chamber, the resorptive soft tissue was removed from the palatal aspect of the crown using Mueller burs (Komet Dental, Brasseler; Lemgo, Germany). The MTA matrix technique was used to restore the palatal aspect of the tooth (Fig 2d). This allowed further treatment using isolation with rubber-dam (Roeko Dental Dam, ROEKO; Langenau, Germany). The remaining root- canal system was cleaned and shaped using nickel-titanium rotary instruments and hand files (VDW; Munich, Germany). ET was accompanied by continuous irrigation, predominantly with sodium hypochlorite 3% (Hedinger; Stuttgart, Germany). The root-canal system was temporarily filled with calcium hydroxide (University Hospital Heidelberg Pharmacy, Germany). After 2 weeks, the open apical part of the root canal was obturated with MTA as an apical plug. Due to the extensive loss of hard tissue, the remaining root-canal system, including the access cavity, was filled with bonded composite resin in very small increments to reduce the risk of fracture.^19,41^ The above mentioned polymerization lamp (VALO-LED curing light, Ultradent) with the adaptable fiber-optics lens (EndoGuide lens for VALO-LED curing light) was used to ensure optimal light polymerization (Fig 2e). The postoperative radiograph revealed a homogeneous root canal filling consisting of composite resin with an apical MTA plug (Fig 2f).

**Fig 2d:**
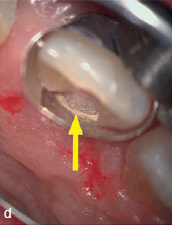
Palatal view after matrix application and sealing of the matrix margin using MTA (arrow).

**Fig 2e:**
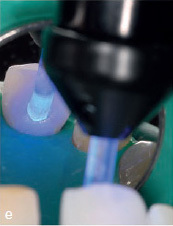
Light polymerization of the palatal composite restoration using a VALO-LED lamp with adapted fiber-optics attachment (EndoGuide lens).

**Fig 2f:**
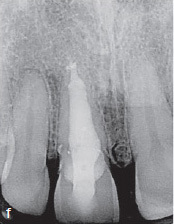
Postoperative radiograph of tooth 11 after root canal filling with an MTA apical plug. The remaining root-canal system is filled with composite resin. The slight surplus of root-canal filling material is MTA.

The clinical and radiographic findings at the follow-up examinations, which were performed at 6 months, 1 and 3 years post-treatment (Figs 2g to 2i), showed no clinical or radiographic pathologic findings and the tooth remained asymptomatic. A CBCT, taken 1 year post-treatment, showed no signs of continuing root resorption, apical pathogenesis, or bone loss at the site of the subgingival composite filling with the epicrestal MTA margin (Fig 2g). Over the entire follow-up period, PDs at the tooth were in the range of 2-4 mm without BOP, indicating healthy periodontal tissues, even at the subgingival filling on the palatal aspect of the tooth (Fig 2i).

**Fig 2 g:**
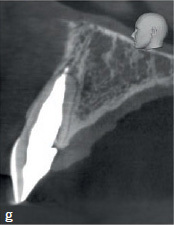
CBCT image (sagittal slice), 1 year post-treatment. Despite the MTA margin of the palatal composite filling being in direct contact with the bone, no radiolucency or bone loss is visible.

**Fig 2h:**
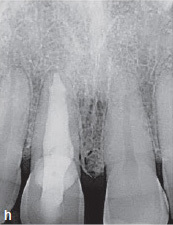
Follow-up radiograph of tooth 11 after 3 years.

**Fig 2i:**
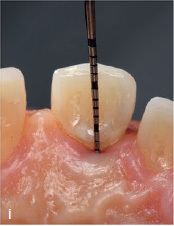
Palatal clinical view at the last follow-up. Pocket depths were between 2–4 mm without BOP over the entire follow-up period.

#### Case 2

A 44-year-old female patient was referred with persistent pain on her maxillary left second molar one month after ET elsewhere. Clinical examination revealed percussion sensitivity. Periodontal probing at the tooth showed an isolated pocket of 6 mm with profuse BOP at the distal aspect. The remaining PDs at this tooth and all PDs at the maxillary left first molar were 2-3 mm without BOP. A CBCT of the area of the tooth, taken before ET by the referring dentist, showed a radiolucent area in the distal cervical area of the maxillary left second molar, consistent with a Heithersay class 3 ECR (Fig 3a).

**Fig 3a:**
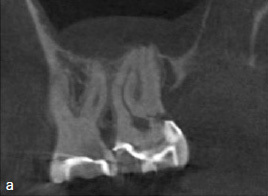
Preoperative sagittal CBCT image of tooth 27 confirmed the presence of a Heithersay class-3 ECR.

Unfortunately, the distal cervical aspect of the perforating resorption (according to the portal of entry of the ECR)^31^ appeared unsealed on the postoperative radiograph taken by the referring dentist (Fig 3b, see arrow).

**Fig 3b:**
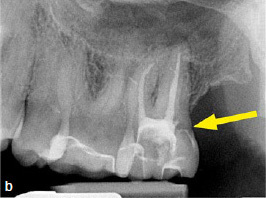
Radiograph of tooth 27, taken by the referring dentist after root-canal filling. The arrow points to the portal of entry of the ECR and appears unsealed.

Based on these clinical and radiographic findings, the patient’s maxillary left second molar was diagnosed as a previously root-canal filled tooth with symptomatic apical periodontitis and an external cervical resorption (Heithersay class 3). The advantages and risks of retreatment with repair of the unsealed area of the perforating resorption were discussed with the patient. The patient opted for an attempt to preserve the tooth.

The pre-endodontic restoration of the perforating resorptive cavity at the distal cervical aspect of the tooth was carried out using the MTA matrix technique. A Tofflemire matrix band (KerrHawe) was inserted and fixed with a matrix band holder (KerrHawe) instead of a rubber-dam clamp. By removing the resorption tissue via the access cavity of the tooth, the distal tooth defect became slightly larger again. The cavity was then filled with MTA at the lower margin and subsequently layered with composite resin. In contrast to case 1, the MTA sealing of the lower margin of the matrix band, and also the subsequent composite application, were performed via the access cavity of the tooth.

As mentioned above in the description of the MTA matrix technique, the MTA cement was compressed with the aid of a moist cotton pellet for a few seconds which prevents the washout of the MTA cement during the subsequent procedures of etching, rinsing, priming, and bonding, before composite application. The matrix band was then removed and replaced by a rubber-dam clamp. Subsequently, retreatment of the old root-filling material using nickel-titanium rotary instruments and hand files (VDW) was performed. The working length radiograph of the tooth after retreatment revealed sufficient repair of the cervical perforation and successful retreatment of the old root-canal filling material (Fig 3c). Treatment was performed in two sessions. Between sessions, the tooth was medicated with a mixture of calcium hydroxide powder and 2% CHX solution (Engelhard Arzneimittel; Niederdorfelden, Germany). Within the second session, MTA apical plugs were placed in all roots. The residual root-canal space was backfilled with injectable gutta-percha (Obtura III, Obtura Spartan; Fenton, MO, USA) and AH Plus sealer (Dentsply Sirona; Konstanz, Germany). The access cavity was sealed with composite resin (Tetric Evo Ceram Bleach XL flow and Tetric Evo Ceram A3 XL, Ivoclar Vivadent), and a postoperative radiograph was taken (Fig 3d).

**Fig 3c:**
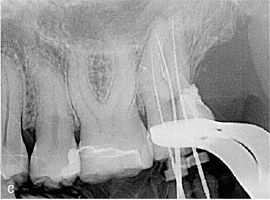
Working length radiograph of tooth 27 after retreatment.

**Fig 3d:**
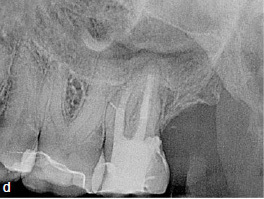
Postoperative radiograph of tooth 27.

The clinical and radiographic findings at the follow-up examinations at 1 year (Fig 3e), 3 years, and 4.5 years (Fig 3f) post-treatment showed no pathological findings. PDs at this maxillary left second molar were 2 to 3 mm. The reduction of the PD from 6 to 3 mm without distal BOP at this tooth indicates complete periodontal healing in the area of the epicrestal hard tissue defect initially caused by the ECR.

**Fig 3e:**
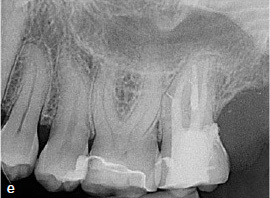
Follow-up radiograph of tooth 27 after 1 year, showing no bone loss close to the epicrestal area where the margin of the restoration consists of MTA.

**Fig 3f:**
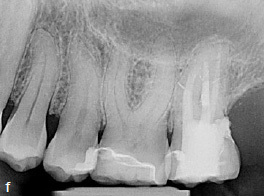
Periapical radiograph of tooth 27 at the 4.5-year follow-up.

#### Case 3

A 45-year-old male patient presented with increasing discomfort around his mandibular left second molar during mastication. He reported that part of the crown of this tooth seemed to be mobile. The tooth did not respond to cold (CO_2_) or electric pulp testing. Clinical examination showed a deep, complicated cusp fracture disto-lingually extending to the osseous crest (Figs 4a and 4b). Periodontal probing showed an isolated pocket of 9 mm distal to the tooth with profuse BOP. The remaining PDs at this tooth and the neighboring first left mandibular molar were 2 to 3 mm. A slightly widened periodontal ligament space at the mesial root and a coronal fracture line reaching down to the epicrestal area were noted upon radiographic examination (Fig 4c). Based on these clinical and radiographic findings, the mandibular left second molar was diagnosed with a disto-lingual cusp fracture extending below the osseous crest with pulpal involvement. The patient was given the options of nonsurgical ET with composite resin restoration of the tooth using the MTA matrix technique, extraction, or intentional replantation. The patient opted for the nonsurgical treatment option. The treatment performed was analogous to the use of the MTA matrix technique in case 1, allowing ET and the composite restoration of the fractured part of the crown to be performed with rubber-dam. The postoperative radiograph showed a homogeneous root-canal filling (Fig 4d). Near the osseous crest, a very thin layer of MTA cement can be identified at the lower end of the composite restoration (Fig 4d).

**Fig 4a:**
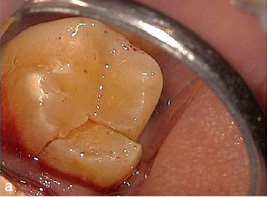
Cusp fracture extending disto-lingually below the osseous crest at tooth 37.

**Fig 4b:**
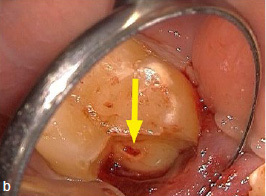
Careful removal of the fractured disto-lingual crown fragment of tooth 37 revealed pulpal involvement (see arrow).

**Fig 4c:**
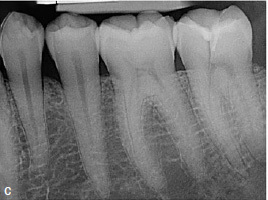
Preoperative radiograph showing the gap caused by the disto-lingual loosening of part of the natural crown of tooth 37 (corresponding to the clinical situation in Figs 4a and 4b).

**Fig 4d:**
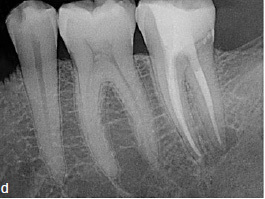
Postoperative radiograph of tooth 37.

Clinical and radiographic follow-up examinations at 3 months, 9 months (Fig 4e), 1.5 years, and 3 years (Fig 4f) posttreatment showed no washout of MTA and no pathological findings, except one single PD of 4 mm at the disto-lingual aspect of this mandibular left second molar without BOP. All other PDs were 2 to 3 mm at all follow-up examinations. The patient was symptom-free without restrictions of masticatory function since the day of the treatment.

**Fig 4e:**
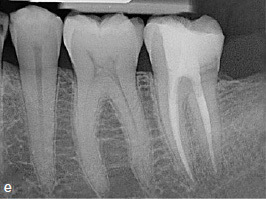
Follow-up radiograph of tooth 37 after 9 months.

**Fig 4f:**
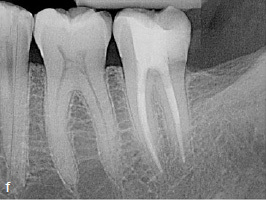
Follow-up radiograph of tooth 37 three years post treatment, showing no bone loss within the area of the very deep margin of the MTA-composite restoration.

## DISCUSSION

The restoration of teeth with deep subgingival hard tissue defects reaching down to the osseous crest poses a challenge to dentists. The described MTA matrix technique can overcome these challenges by enabling dry conditions for composite restorations when optimal matrix adaptation is not achievable. It allows tight adaption of the matrix band in cases of extremely deep cavities. The MTA provides a biocompatible layer in direct contact with the crestal bone tissue, acts as a barrier to fluids, and is even able to compensate for a matrix margin that is too short (Figs 1b and 1c). If the MTA cement is compressed into the small gap between the lower end of the matrix and the bone crest with the aid of a moist cotton pellet for a few seconds, it will prevent washout of MTA during the subsequent procedure of etching, rinsing, priming, and bonding. Immediate placement of composite resin on top of the compressed MTA cement is thereby possible directly afterwards.

The question could be raised whether the basic pH of the MTA cement during setting may potentially affect the quality of the adhesive bond to the dentin, because the composite is applied on non-set MTA. This question of the best timing for composite resin placement on MTA was evaluated by Tsujimoto et al,^39^ who examined MTA/composite interfaces after different restoration timepoints, as well as with and without a self-etch bonding agent.^39^ The findings of this study support the immediate placement of composite resin with a bonding agent on top of non-set MTA within a single visit. The findings of this study support the immediate placement of composite resin with a bonding agent onto non-set MTA in a single visit.

With the MTA matrix technique, no ferrule effect can be achieved in a subsequent post-endodontic restoration of the tooth with a crown. However, there are other prosthetic restoration concepts, eg, a partial crown or endocrown, for which no ferrule effect is required.^2,12,25^ When using the MTA matrix technique, it is impossible to respect STA. Surgical measures to avoid the violation of STA, such as surgical crown lengthening or removal of oral mucosa around deep cavities, can lead to black triangles or other adverse esthetic or periodontal consequences.^4,15,37^ Orthodontic extrusion provides improved esthetic results, but is more expensive and time consuming. This type of extrusion is usually performed in multiple appointments over several weeks and a 2-3 months retention period is required to stabilize the tooth in the extruded position.^10,34^

Violation of STA is usually associated with gingival inflammation, loss of periodontal attachment, and localized bone loss, and is therefore regarded as a risk factor in restoration of teeth with deep cavities.^13,30,36^ A particularly interesting study in this context is the prospective clinical study by Dragoo et al.^8^ The authors examined two resin ionomers and one hybrid ionomer for the restoration of 50 subgingival dental lesions followed by histological analysis. The histological findings suggested adherence of epithelium and connective tissue to the resin ionomers.^8^ Regarding the use of composites, a more recent prospective clinical study evaluated the clinical and histological response of the supracrestal periodontal tissues to subgingival composite restorations 3 months post-treatment vs untreated root surfaces.^3^ The results suggest that well-shaped and well-refined subgingival composite restorations resulted in soft-tissue health similar to natural root surfaces.^3^

To overcome the technical difficulties in the restoration of extremely deep and undermining tooth defects and to allow appropriate rubber-dam isolation for moisture control for the placement of direct or indirect restorations under clinically manageable conditions, treatment techniques such as cervical margin relocation,^7^ deep margin elevation,^3,5,27,35^ R2-technique,^11^ or proximal box elevation^21,32^ have been introduced and have shown encouraging clinical and histological results.^3,5^ The dogma that the supracrestal tissue attachment must be preserved at all times is questioned by the findings of some clinical studies and case series. These elevation techniques resulted in stable and at least not worsened periodontal conditions when compared to cases in which surgical crown lengthening was performed.^11,28,35^

As a supplement to these elevation techniques, the MTA matrix technique allows restoration of even deeper hard tissue defects extending to the osseous crest. The authors have not yet observed any unfavorable reaction of the periodontal tissues to the MTA margins. Case 1 shows that not even the overhanging restoration margin in direct bone contact leads to bone recession or osteolysis. Due to its biocompatibility, MTA does not seem to cause any reaction of the periodontal tissues.

In contrast to sandwich techniques, about which various in-vitro studies,^1,22^ case reports^23^ and clinical trials^26^ have been published repeatedly for many years, MTA used with the MTA matrix technique does not serve as a dentin substitute. It is not layered on the whole cavity floor or used as a protective layer for the pulp, but rather serves as an extension of the matrix band (which could not be inserted to a sufficient depth), creating a barrier that reliably prevents the ingress of blood and sulcular fluid. This is illustrated by Fig 1c (the area outlined in red) and Fig 2d (yellow arrow). This allows the dry and thus sufficient application of composite resin, which is superior to calcium-silicate cements such as MTA or Biodentine in terms of bonding to dentin under dry conditions.^20^

## CONCLUSION

The clinical findings of the three exemplary cases presented are not yet sufficient to generally recommend the use of the MTA matrix technique. However, this technique seems to be a feasible treatment option for teeth with deep subgingival tooth defects, and well worth considering. Clinical studies, especially prospective ones, are required to confirm the long-term feasibility and success rates of this technique. A clinical trial is already in progress at the authors’ university hospital.

## Clinical relevance:

The MTA matrix technique is an alternative technique to restore teeth with deep, subgingival, hard tissue defects extending down to the osseous crest. It combines mineral trioxide aggregate (MTA) and composite resin without requiring surgical or orthodontic interventions.

## References

[ref1] Aggarwal V, Singla M, Yadav S, Yadav H, Ragini (2015). Marginal adaptation evaluation of Biodentine and MTA Plus in “open sandwich” class II restorations. J Esthet Restor Dent.

[ref2] Belleflamme MM, Geerts SO, Louwette MM, Grenade CF, Vanheusden AJ, Mainjot AK (2017). No post-no core approach to restore severely damaged posterior teeth: An up to 10-year retrospective study of documented endocrown cases. J Dent.

[ref3] Bertoldi C, Monari E, Cortellini P, Generali L, Lucchi A, Spinato S, Zaffe D (2020). Clinical and histological reaction of periodontal tissues to subgingival resin composite restorations. Clin Oral Investig.

[ref4] Bragger U, Lauchenauer D, Lang NP (1992). Surgical lengthening of the clinical crown. J Clin Periodontol.

[ref5] Bresser RA, Gerdolle D, van den Heijkant IA, Sluiter-Pouwels LMA, Cune MS, Gresnigt MMM (2019). Up to 12 years clinical evaluation of 197 partial indirect restorations with deep margin elevation in the posterior region. J Dent.

[ref6] Das B, Muthu MS (2013). Surgical extrusion as a treatment option for crown-root fracture in permanent anterior teeth: a systematic review. Dent Traumatol.

[ref7] Dietschi D, Spreafico R (1998). Current clinical concepts for adhesive cementation of tooth-colored posterior restorations. PPAD.

[ref8] Dragoo MR (1997). Resin-ionomer and hybrid-ionomer cements: part II, human clinical and histologic wound healing responses in specific periodontal lesions. Int J Periodontics Restorative Dent.

[ref9] Elkhadem A, Mickan S, Richards D (2014). Adverse events of surgical extrusion in treatment for crown-root and cervical root fractures: a systematic review of case series/reports. Dent Traumatol.

[ref10] Faria LP, Almeida MM, Amaral MF, Pellizzer EP, Okamoto R, Mendonca MR (2015). Orthodontic extrusion as treatment option for crown-root fracture: Literature review with systematic criteria. J Contemp Dent Pract.

[ref11] Frese C, Wolff D, Staehle HJ (2014). Proximal box elevation with resin composite and the dogma of biological width: clinical R2-technique and critical review. Oper Dent.

[ref12] Govare N, Contrepois M (2020). Endocrowns: A systematic review. J Prosthet Dent.

[ref13] Günay H, Seeger A, Tschernitschek H, Geurtsen W (2000). Placement of the preparation line and periodontal health –a prospective 2-year clinical study. Int J Periodont Restor Dent.

[ref14] Heithersay GS (1999). Invasive cervical resorption: an analysis of potential predisposing factors. Quintessence Int.

[ref15] Hempton TJ, Dominici JT (2010). Contemporary crown-lengthening therapy: a review. J Am Dent Assoc.

[ref16] Ingber JS (1976). Forced eruption: part II. A method of treating nonrestorable teeth –Periodontal and restorative considerations. J Periodontol.

[ref17] Jepsen S, Caton JG, Albandar JM, Bissada NF, Bouchard P, Cortellini P, Demirel K, de Sanctis M, Ercoli C, de Sanctis M, Fan J, Geurs NC, Hughes FJ, Jin L, Kantarci A, Lalla E, Madianos PN, Matthews D, McGuire MK, Mills MP, Preshaw PM, Reynolds MA, Sculean A, Susin C, West NX, Yamazaki K (2018). Periodontal manifestations of systemic diseases and developmental and acquired conditions: Consensus report of workgroup 3 of the 2017 World workshop on the classification of periodontal and peri-implant diseases and conditions. J Clin Periodontol.

[ref18] Kahnberg KE (1988). Surgical extrusion of root-fractured teeth –a follow-up study of two surgical methods. Endod Dent Traumatol.

[ref19] Karapinar-Kazandag M, Basrani B, Tom-Kun Yamagishi V, Azarpazhooh A, Friedman S (2016). Fracture resistance of simulated immature tooth roots reinforced with MTA or restorative materials. Dent Traumatol.

[ref20] Kaup M, Dammann CH, Schafer E, Dammaschke T (2015). Shear bond strength of Biodentine, ProRoot MTA, glass ionomer cement and composite resin on human dentine ex vivo. Head Face Med.

[ref21] Kielbassa AM, Philipp F (2015). Restoring proximal cavities of molars using the proximal box elevation technique: Systematic review and report of a case. Quintessence Int.

[ref22] Koubi S, Elmerini H, Koubi G, Tassery H, Camps J (2012). Quantitative evaluation by glucose diffusion of microleakage in aged calcium silicate-based open-sandwich restorations. Int J Dent.

[ref23] Kqiku L, Ebeleseder KA, Glockner K (2012). Treatment of invasive cervical resorption with sandwich technique using mineral trioxide aggregate: a case report. Oper Dent.

[ref24] Krug R, Connert T, Soliman S, Syfrig B, Dietrich T, Krastl G (2018). Surgical extrusion with an atraumatic extraction system: A clinical study. J Prosthet Dent.

[ref25] Li X, Kang T, Zhan D, Xie J, Guo L (2020). Biomechanical behavior of endocrowns vs fiber post-core-crown vs cast post-core-crown for the restoration of maxillary central incisors with 1 mm and 2 mm ferrule height: A 3D static linear finite element analysis. Medicine (Baltimore).

[ref26] Lindberg A, van Dijken JW, Lindberg M (2003). 3-year evaluation of a new open sandwich technique in class II cavities. Am J Dent.

[ref27] Magne P, Spreafico RC (2012). Deep margin elevation: a paradigm shift. Am J Esthet Dent.

[ref28] Oppermann RV, Gomes SC, Cavagni J, Cayana EG, Conceicao EN (2016). Response to proximal pestorations placed either subgingivally or following crown lengthening in patients with no history of periodontal disease. Int J Periodontics Restorative Dent.

[ref29] Pama-Benfenati S, Fugazzotto PA, Ferreira PM, Ruben MP, Kramer GM (1986). The effect of restorative margins on the postsurgical development and nature of the periodontium. Part II Anatomical considerations. Int J Periodont Restor Dent.

[ref30] Paniz G, Nart J, Gobbato L, Mazzocco F, Stellini E, De Simone G, Bressan E (2017). Clinical periodontal response to anterior all-ceramic crowns with either chamfer or feather-edge subgingival tooth preparations: Six-month results and patient perception. Int J Periodontics Restorative Dent.

[ref31] Patel S, Mavridou AM, Lambrechts P, Saberi N (2018). External cervical resorption-part 1: histopathology, distribution and presentation. Int Endod J.

[ref32] Roggendorf MJ, Kramer N, Dippold C, Vosen VE, Naumann M, Jablonski-Momeni A, Frankenberger R (2012). Effect of proximal box elevation with resin composite on marginal quality of resin composite inlays in vitro. J Dent.

[ref33] Roth A, Yildirim M, Diedrich P (2004). Forced eruption with microscrew anchorage for preprosthetic leveling of the gingival margin Case report. J Orofac Orthop.

[ref34] Saito CT, Guskuma MH, Gulinelli JL, Sonoda CK, Garcia-Junior IR, Filho OM, Panzarini SR (2009). Management of a complicated crown-root fracture using adhesive fragment reattachment and orthodontic extrusion. Dent Traumatol.

[ref35] Sarfati A, Tirlet G (2018). Deep margin elevation versus crown lengthening: biologic width revisited. Int J Esthet Dent.

[ref36] Tal H, Soldinger M, Dreiangel A, Pitaru S (1989). Periodontal response to long-term abuse of the gingival attachment by supracrestal amalgam restorations. J Clin Periodontol.

[ref37] Tarnow DP, Magner AW, Fletcher P (1992). The effect of the distance from the contact point to the crest of bone on the presence or absence of the interproximal dental papilla. J Periodontol.

[ref38] Toure B, Faye B, Kane AW, Lo CM, Niang B, Boucher Y (2011). Analysis of reasons for extraction of endodontically treated teeth: a prospective study. J Endod.

[ref39] Tsujimoto M, Tsujimoto Y, Ookubo A, Shiraishi T, Watanabe I, Yamada S, Hayashi Y (2013). Timing for composite resin placement on mineral trioxide aggregate. J Endod.

[ref40] Tzimpoulas NE, Alisafis MG, Tzanetakis GN, Kontakiotis EG (2012). A prospective study of the extraction and retention incidence of endodontically treated teeth with uncertain prognosis after endodontic referral. J Endod.

[ref41] Wilkinson KL, Beeson TJ, Kirkpatrick TC (2007). Fracture resistance of simulated immature teeth filled with resilon, gutta-percha, or composite. J Endod.

[ref42] Zadik Y, Sandler V, Bechor R, Salehrabi R (2008). Analysis of factors related to extraction of endodontically treated teeth. Oral Surg Oral Med Oral Pathol Oral Radiol Endod.

